# Supplementary material for: Chemistry-intuitive explanation of graph neural networks for molecular property prediction with substructure masking
Source: Nat Commun. 2023 May 4;14:2585. doi: 10.1038/s41467-023-38192-3 (PMC10160109; doi:10.1038/s41467-023-38192-3)
Supplement: Supplementary file 1 — Supplementary Information [file 41467_2023_38192_MOESM1_ESM.pdf]

Supplementary Information

Chemistry-intuitive explanation of graph neural networks for molecular property prediction with substructure masking

Zhenxing Wu[1,2], Jike Wang[1,2,3], Hongyan Du[1,2], Dejun Jiang[1,2], Yu Kang[1], Dan Li[1], Peichen Pan[1], Yafeng Deng[2], Dongsheng Cao[4,*], Chang-Yu Hsieh[1,*], Tingjun Hou[1,*]

[1]Innovation Institute for Artificial Intelligence in Medicine of Zhejiang University, College of Pharmaceutical Sciences, Zhejiang University, Hangzhou 310058, Zhejiang, P. R. China

[2]CarbonSilicon AI Technology Co., Ltd, Hangzhou 310018, Zhejiang, P. R. China

[3]National Engineering Research Center for Multimedia Software, School of Computer Science, Wuhan University, Wuhan 430072, Hubei, P. R. China.

[4]Xiangya School of Pharmaceutical Sciences, Central South University, Changsha 410004, Hunan, P. R. China

Supplementary Table 1. The performance of the consensus models on the test sets.

Model	Type	Metric	Performance
BBBP_MW	regression	R^2	0.9906
BBBP_LogP	regression	R^2	0.9829
BBBP_TPSA	regression	R^2	0.9997
BBBP_HBDs	regression	R^2	0.9923

Supplementary Table 2. The details of the four datasets.

Dataset	Type	Data capacity	Positive sample size	Negative sample size
ESOL	regression	1111	—	—
Mutagenicity	classification	7672	Mutagens: 4309	Nonmutagens: 3363
hERG	classification	9876	Bloockers: 5090	Nonblockers: 4786
BBBP	classification	1859	BBB+: 1433	BBB-: 426

Supplementary Table 3. The detailed information of different datasets.

**Category**	**Description**	**The number of molecules**
ESOL	Small dataset consisting of water solubility data for 1111 compounds[1]. The duplicated molecules and the molecules with conflicting label values are excluded.	1111
Mutagenicity	The training set for model building was collected from four papers. The data set for external validation was extracted from the Web site of Lazar toxicity predictions. The entire database was prepared as following. First, apart from the four false SMILES strings, duplicate molecules were removed from the five sources by using canonical SMILES. Second, molecules without clear E or Z configuration were removed. Third, inorganic compounds were omitted from the data set.	7672

	The last step was to eliminate the tautomers and compounds with molecular weight less than 40 or more than 800 in the data set. When doing the data set curation, we followed one principle. For a given compound, if the experimental mutagenicity data varied in different sources, the compound was cleared out. For compounds without defined steric configuration or tautomers, if the experimental mutagenicity data was alike, then only one structure was kept, and the others were deleted.[2]	
hERG	The original chemicals with experimental IC50 values are collected from a publication[3] and CHEMBL database. Molecules with IC50 ≤10 μM are classified as hERG blockers, and molecules with IC50 > 10μM are classified as hERG nonblockers. Inorganic compounds, noncovalent complexes and mixtures are removed from the data set. The duplicated molecules and the molecules with conflicting label values were excluded.	9876
BBBP	The dataset is from ADMET lab 2.0.[4]<br>Category 0: BBB-; Category 1: BBB+;<br>The molecules were divided into BBB+ and BBB-classes with logBB ≥-1 and logBB < -1, respectively.	1859

Supplementary Table 4. The canonical SMILES of the compounds for analysis.

Molecule	Canonical SMILES
**Compound 1**	CC1CCC(C(C1)O)C(C)C
**Compound 2**	COc1ccc(C(O)(c2cncnc2)C2CC2)cc1
**Compound 3**	Nc1c(C(=O)O)cc([N+](=O)[O-])c2c1C(=O)c1ccccc1C2=O
**Compound 4**	Cc1ccc(N)cc1[N+](=O)[O-]
**Compound 5**	COc1cc([N+](=O)[O-])ccc1N

**Compound 6**	[N-]=[N+]=Nc1ccc(F)c([N+](=O)[O-])c1
**Compound 7**	O=[N+](c1cc2c(cccc2)c2ccccc21)[O-]
**Compound 8**	NCc1ccc(F)c(C2CCN(C(=O)c3cccc(-c4nc(-c5cccs5)no4)c3)CC2)c1
**Compound 9**	NCc1ccc(F)c(C2CCN(C(=O)c3cc(C(=O)O)cc(-c4nc(-c5cccs5)no4)c3)CC2)c1
**Compound 10**	NCc1ccc(F)c(C2CCN(C(=O)c3cc(C(N)=O)cc(-c4nc(-c5cccs5)no4)c3)CC2)c1
**Compound 11**	COc1ccc(CCN2CCC(CCc3ccccc3OCCF)CC2)cc1
**Compound 12**	FCCOc1ccccc1CCC1CCN(CCc2ccccc2)CC1
**Compound 13**	FCCOc1ccccc1CCN1CCN(CCc2ccccc2Cl)CC1
**Compound 14**	FCCOc1ccccc1CCN1CCN(CCc2ccccc2)CC1
**Compound 15**	CCn1nc(Cc2ccc(C#N)cc2)cc1C1CCN(C[C@H]2CN([C@@H](C(=O)O)C(C)(C)C)C[C@@H]2c2cccc(F)c2)CC1
**Compound 16**	CCn1nc(Cc2ccc(S(C)(=O)=O)cc2)cc1C1CCN(C[C@H]2CN([C@@H](C(=O)O)C(C)(C)C)C[C@@H]2c2cccc(F)c2)CC1
**Compound 17**	Nc1ccc2nc(Cc3ccc(Oc4ccccc4)cc3)[nH]c2c1
**Compound 18**	CC(=O)Nc1ccc2nc(Cc3ccc(Oc4ccccc4)cc3)[nH]c2c1

Supplementary Table 5. The initial node (atom) and edge (bond) information used in RGCN.

Node(atom) feature	Size	Description
**Atom symbol**	16	[B, C, N, O, F, Si, P, S, Cl, As, Se, Br, Te, I, At, metal] (one-hot)
**degree**	6	number of covalent bonds [0,1,2,3,4,5] (one-hot)
**formal charge**	1	electrical charge (integer)
**hybridization**	6	[sp, sp2, sp3, sp3d, sp3d2, other] (one-hot)
**aromaticity**	1	whether the atom is part of an aromatic system [0/1] (one-hot)
**hydrogens**	5	number of connected hydrogens [0,1,2,3,4] (one-hot)
**chirality**	1	whether the atom is chiral center [0/1] (one-hot)
**chirality type**	2	[R, S] (one-hot)

Edge (bond) feature	Size	Description
**bond type**	4	[single, double, triple, aromatic]
**conjugation**	1	whether the bond is conjugated [0/1]
**ring**	1	whether the bond is in ring [0/1]
**stereo**	4	[StereoNone, StereoAny, StereoZ, StereoE]

Supplementary Table 6. The hyperparameters of different models.

Model	Parameters to be optimized	Package
ESOL	the number of nodes of each RGCN hidden layer: [64, 128, **256**]<br>the number of RGCN hidden layer: [**2**, 3]<br>the number of nodes of each FC hidden layer: [**64**, 128, 256]<br>the dropout rate of each RGCN hidden layer: [0, 0.1, 0.2, 0.3, 0.4, **0.5**]<br>the dropout rate of each FC hidden layer: [0, **0.1**, 0.2, 0.3, 0.4, 0.5]<br>the learning rate: [**0.003**, 0.001, 0.0003, 0.0001]<br>the number of epochs: 500<br>the patience of early stop: 30	DGL 0.7.1
Mutagenicity	the number of nodes of each RGCN hidden layer: [64, 128, **256**]<br>the number of RGCN hidden layer: [2, **3**]<br>the number of nodes of each FC hidden layer: [64, **128**, 256]<br>the dropout rate of each RGCN hidden layer: [0, 0.1, 0.2, 0.3, **0.4**, 0.5]<br>the dropout rate of each FC hidden layer: [**0**, 0.1, 0.2, 0.3, 0.4, 0.5]	DGL 0.7.1

	the learning rate: [0.003, **0.001**, 0.0003, 0.0001]<br>the number of epochs: 500<br>the patience of early stop: 30	
hERG	the number of nodes of each RGCN hidden layer: [**64**, 128, 256]<br>the number of RGCN hidden layer: [2, **3**]<br>the number of nodes of each FC hidden layer: [64, **128**, 256]<br>the dropout rate of each RGCN hidden layer: [0, 0.1, **0.2**, 0.3, 0.4, 0.5]<br>the dropout rate of each FC hidden layer: [0, **0.1**, 0.2, 0.3, 0.4, 0.5]<br>the learning rate: [0.003, 0.001, **0.0003**, 0.0001]<br>the number of epochs: 500<br>the patience of early stop: 30	DGL 0.7.1
BBBP	the number of nodes of each RGCN hidden layer: [64, 128, **256**]<br>the number of RGCN hidden layer: [**2**, 3]<br>the number of nodes of each FC hidden layer: [64, **128**, 256]<br>the dropout rate of each RGCN hidden layer: [0, 0.1, **0.2**, 0.3, 0.4, 0.5]<br>the dropout rate of each FC hidden layer: [0, 0.1, 0.2, 0.3, **0.4**, 0.5]<br>the learning rate: [0.003, **0.001**, 0.0003, 0.0001]<br>the number of epochs: 500<br>the patience of early stop: 30	DGL 0.7.1

Bold hyperparameters represent optimized hyperparameters.

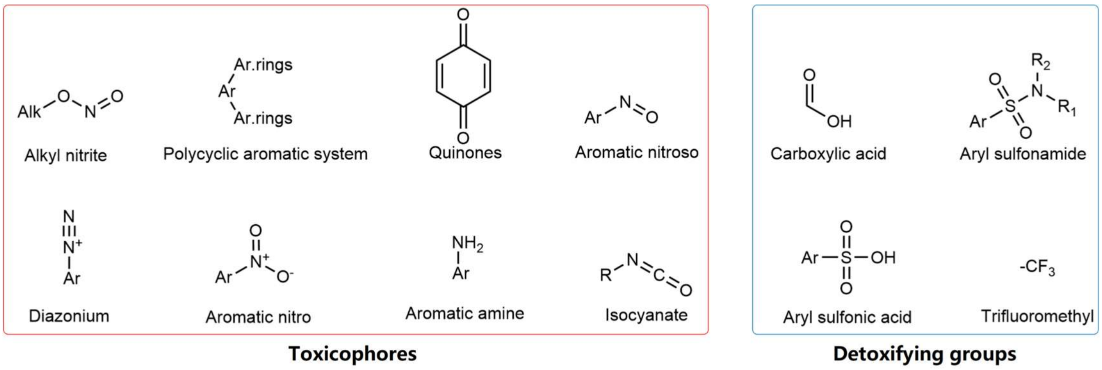

Supplementary Figure 1. Some identified toxicophores and detoxifying groups.[2, 5-8] 'Ar' indicates an aromatic atom, 'Alk' indicates an alkyl atom, and 'Ar.rings' indicates an atom that is part of multiple aromatic rings.

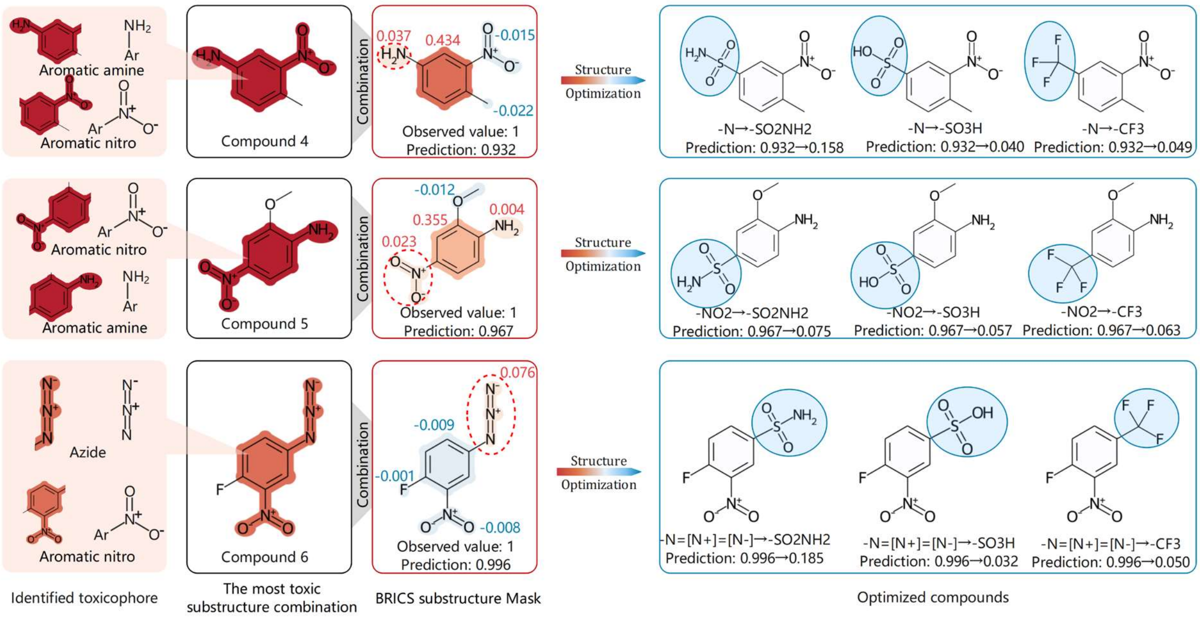

Supplementary Figure 2. The attribution visualization and structural optimization of **compounds 4**, **5** and **6**; The toxic functional groups in **compounds 4**, **5**, and **6** (the amino, nitro and isocyanate groups) are changed to detoxifying groups (sulfonyl hydroxide, sulfonamide, and trifluoromethyl).

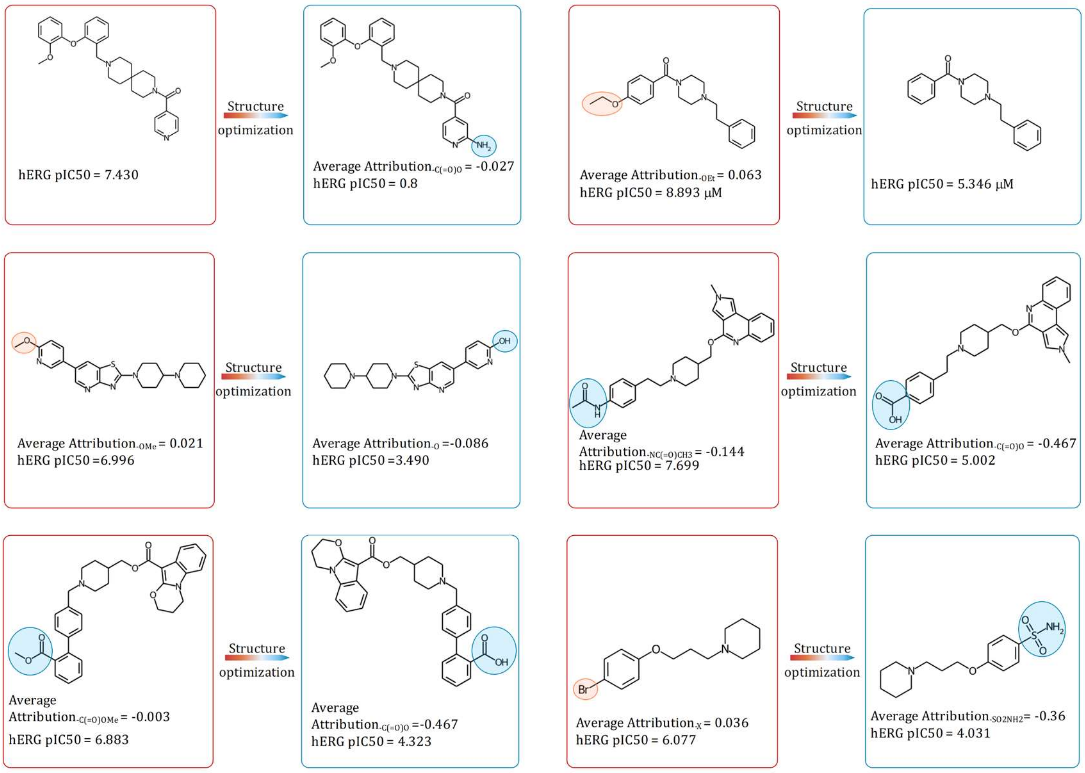

Supplementary Figure 3. Some real-world hERG cliff molecular pairs of hERG toxicity.

Supplementary References

1. Mobley, D. L.; Guthrie, J. P., FreeSolv: a database of experimental and calculated hydration free energies, with input files. *Journal of computer-aided molecular design* **2014,** *28* (7), 711-720.
2. Xu, C.; Cheng, F.; Chen, L.; Du, Z.; Li, W.; Liu, G.; Lee, P. W.; Tang, Y., In silico prediction of chemical Ames mutagenicity. *Journal of Chemical Information and Modeling* **2012,** *52* (11), 2840-2847.
3. Zhang, C.; Zhou, Y.; Gu, S.; Wu, Z.; Wu, W.; Liu, C.; Wang, K.; Liu, G.; Li, W.; Lee, P. W., In silico prediction of hERG potassium channel blockage by chemical category approaches. *Toxicology research* **2016,** *5* (2), 570-582.
4. Xiong, G.; Wu, Z.; Yi, J.; Fu, L.; Yang, Z.; Hsieh, C.; Yin, M.; Zeng, X.; Wu, C.; Lu, A., ADMETlab 2.0: an integrated online platform for accurate and comprehensive predictions of ADMET properties. *Nucleic Acids Research* **2021,** *49* (W1), W5-W14.
5. Wu, Z.; Jiang, D.; Wang, J.; Hsieh, C.-Y.; Cao, D.; Hou, T., Mining toxicity information from large amounts of toxicity data. *Journal of Medicinal Chemistry* **2021,** *64* (10), 6924-6936.
6. Bakhtyari, N. G.; Raitano, G.; Benfenati, E.; Martin, T.; Young, D., Comparison of in silico models for prediction of mutagenicity. *Journal of Environmental Science and Health, Part C* **2013,** *31* (1), 45-66.

7. Hansen, K.; Mika, S.; Schroeter, T.; Sutter, A.; Ter Laak, A.; Steger-Hartmann, T.; Heinrich, N.; Muller, K.-R., Benchmark data set for in silico prediction of Ames mutagenicity. *Journal of chemical information and modeling* **2009,** *49* (9), 2077-2081.
8. Polishchuk, P. G.; Kuz'min, V. E.; Artemenko, A. G.; Muratov, E. N., Universal approach for structural interpretation of QSAR/QSPR models. *Molecular Informatics* **2013,** *32* (9 - 10), 843-853.
