# Supplementary material for: Chemistry-intuitive explanation of graph neural networks for molecular property prediction with substructure masking
Source: Nat Commun. 2023 May 4;14:2585. doi: 10.1038/s41467-023-38192-3 (PMC10160109; doi:10.1038/s41467-023-38192-3)
Supplement: Supplementary file 3 — Description of Additional Supplementary Files [file 41467_2023_38192_MOESM3_ESM.pdf]

Description of Additional Supplementary Files

File Name: Supplementary Data 1
Description: Details on the structural optimization of compound 1, this data table provides the source data of Figure 2. The table includes data on the functional group that was optimized (methyl group), the SMILES of compound 1, the average attribution of the functional group, and the predicted value of compound 1using the ESOL model. The table also presents information on the optimized molecule, including the optimized functional group, the SMILES of the optimized molecule, the attribution of the optimized functional group, and the predicted values of the optimized molecule by the ESOL model.

File Name: Supplementary Data 2
Description: The correlation of the average attributions between hERG and ESOL's functional groups as discussed in Figure 7. This table provides the source data, and the average attribution of various functional groups based on the prediction model for hERG and ESOL mentioned in the main text.

File Name: Supplementary Data 3
Description: The relationship between BBBP and the physicochemical properties. This table provides the source data of **Figure 10**. This table presents the average attribution of various functional groups based on BBBP, BBBP_MW, BBBP_LogP, BBBP_TPSA, and BBBP_HBDs models, which establishes the relationship between BBBP and physicochemical properties.
